# A retrospective study of the clinical characteristics of 9 children with pulmonary embolism associated with Mycoplasma pneumoniae pneumonia

**DOI:** 10.1186/s12887-023-04188-7

**Published:** 2023-07-20

**Authors:** Shaoxiu Song, Yongsheng Xu

**Affiliations:** Department of Pulmonology, Tianjin Children’s Hospital (Children’s Hospital of Tianjin University), Tianjin, China

**Keywords:** Children, Mycoplasma pneumoniae pneumonia, Pulmonary embolism, Rivaroxaban

## Abstract

**Objective:**

The aim of this study was to analyze the clinical characteristics and treatment of children with Mycoplasma pneumoniae pneumonia (MPP) who also present with pulmonary embolism (PE).

**Methods:**

This retrospective analysis examined the demographic data, clinical manifestations, laboratory tests, imaging characteristics, therapy, and prognosis of nine cases of children with Mycoplasma pneumoniae pneumonia (MPP) complicated by pulmonary embolism (PE). The study focused on patients admitted to the respiratory department of Tianjin Children’s Hospital between January 2018 and December 2021.

**Results:**

The age range of the patients was 3 to 8 years old, with a median age of 7.5 years. The median number of days from pulmonary infection to the diagnosis of embolism was 14 days. All patients had refractory Mycoplasma pneumoniae pneumonia (RMPP). Among them, three patients reported chest pain, one of whom had hemoptysis, while five patients had dyspnea, and six patients experienced radiating pain at unusual sites. Five out of the nine children tested positive for lupus anticoagulant (LA), five for anticardiolipin antibody (ACA), three for anti-2-glycoprotein antibody IgM, four for reduced protein S or protein C activity, and three for elevated coagulation factor VIII. Moreover, six out of the nine children tested positive for antinuclear antibodies. All the children underwent CT pulmonary angiograms, which revealed filling defects. After sequential low-molecular heparin anticoagulation with rivaroxaban, nine children in this study showed a good prognosis, with two of them receiving thrombolytic therapy for combined cardiac embolism. Follow-up at 0.5-9 months showed the gradual resolution of the emboli in all 9 children, with no thrombotic recurrences and normalized autoantibodies and thrombophilia markers.

**Conclusions:**

The majority of cases involving Mycoplasma pneumoniae pneumonia (MPP) combined with pulmonary embolism (PE) were diagnosed with refractory MPP (RMPP). However, PE did not always occur in the advanced stages of the disease. Most patients presented with transient autoantibody positivity, abnormal coagulation, and fibrinolytic balance. With timely treatment, the prognosis of MPP combined with PE is generally good. Additionally, rivaroxaban treatment has been shown to be safe and effective.

## Introduction

Pulmonary embolism (PE) is a group of diseases or clinical syndromes caused by various types of emboli that block the pulmonary arteries and their branches. These emboli can be classified as thrombotic pulmonary embolism, amniotic pulmonary embolism, fatty pulmonary embolism, air embolism, tumor embolism, and bacterial embolism. The most common type of PE is thrombotic pulmonary embolism. While pulmonary embolism is mostly reported in adults, cases of PE in children are rare. Previous literature suggests that the incidence of acute PE in hospitalized children ranges from 8.6 to 57 per 100,000 [1, 2]. In community children, the incidence is estimated to be between 0.14 and 0.9 per 100,000 [3, 4]. Unfortunately, there is no accurate epidemiological data related to PE in children in China.

Mycoplasma pneumoniae (MP) is a common cause of community-acquired pneumonia (CAP) in children [5, 6], accounting for 10-40% of CAP cases in hospitalized children. While MP infection is typically limited to the respiratory system and is self-limiting, it can also affect all organ systems. The clinical manifestations of MP infection, combined with Pulmonary Embolism (PE), are atypical and can easily be confused with the underlying disease. According to literature, 16% of children with PE do not exhibit typical symptoms [1], which often leads to delayed diagnosis. In this study, we retrospectively analyzed the general conditions, clinical manifestations, ancillary examinations, and treatment of 9 children with MPP combined with PE, and summarized the clinical characteristics related to MP in children, with the aim of improving the understanding, diagnosis, and treatment of PE related to MPP.

## Methods

### Study population

In the medical record system, we identified 16 confirmed cases of pulmonary embolism (PE) using discharge records and disease ICD codes, while 7 children with PE were excluded due to other diseases. Our research focused on a total of nine children with both PE and MPP. We analyzed their age, sex, disease duration, time of embolism, clinical manifestations, plasma D-dimer, fibrinogen (FIB), PLT count, C-reactive protein (CRP), interleukin-6 (IL-6), antiphospholipid antibody (aPL), antinuclear antibody (ANA), imaging findings, treatment plan, and regression. The study was approved by the Ethics Committee of Tianjin Children’s Hospital, and all subjects’ medical records were reviewed anonymously.

### Diagnostic criteria

The diagnostic criteria for MPP were defined as follows [7]: (1) clinical manifestations of fever and cough; (2) chest imaging showing infiltrates; and (3) a serum anti-MP IgM titer of 1:160 or a four-fold rise in titer in acute and convalescent serum specimens or PCR test MP-RNA positivity. The diagnosis of thrombotic diseases was confirmed through CT pulmonary angiography. Exclusion criteria included: (1) other causes of pulmonary embolism, such as air embolism, PE due to congenital heart disease, central venous catheterization, nephrotic syndrome, surgery, tumor, and adolescent girls taking oral contraceptives; and (2) cases with insufficient clinical data.

### Statistical analyses

For the descriptive analysis, count data were presented as the number of cases, while measurement data were reported as ranges.

## Results

### Baseline data

There were nine children diagnosed with RMPP, which is characterized by a high fever that does not subside or progressive changes on imaging despite conventional use of macrolide antibiotics for over 7 days, all of whom had both RMPP and PE. The group consisted of four males and five females, with ages ranging from 3 to 8 years and a median age of 7.5 years. The median number of days from onset of MPP to diagnosis of PE was 14 days. None of these children had a family history of thrombophilia or hereditary disease.

### Clinical characteristics of patients

At the time of diagnosis of MPP, children mainly presented with cough and fever. Symptoms of embolisms appeared in six children after their fever had subsided and cough had improved. At the time of diagnosis of PE, chest pain occurred in three cases, hemoptysis in one case, dyspnea in five cases, shortness of breath in three cases, hypoxemia in three cases, neck pain in one case, shoulder pain in two cases, subxiphoid pain in one case, pain in the right rib quadrant in one case, and abdominal pain in one case. Two cases showed cyanosis of the lips. Due to massive pleural effusion, three cases had a positive trismus sign, and decreased breath sounds were present in all nine cases. None of the 9 children had symptoms of deep vein thrombosis such as swelling and pain in the lower extremities.

### Laboratory data

Two of our nine children had EBV co-infection, but none had bacterial co-infection. When PE was diagnosed, all 9 children had elevated D-dimer, the median D-dimer was 6.3 (8.91) mg/L(reference value 0-0.3 mg/L), the median fibrinogen (FIB) was 5.288 (2.099) g/L(reference value 1.8-4.0 g/L), the median platelet (PLT) count was 316 (108)×10^9^ /L(reference value (100–300)×10^9^ /L), the median C-reactive protein (CRP) was 122.5 (129.7) mg/L(reference value 0–8 mg/L), and the median interleukin-6 (IL-6) level was 56.36 (95.66) ng/L(reference value 0–7 ng/L). All nine children underwent testing for thrombophilia-related indicators, including antiphospholipid antibodies (aPL), protein C and protein S activity, antibodies to coagulation factors, and coagulation factor inhibitors. Five of them tested positive for lupus anticoagulant (LA), five were positive for anti-cardiolipin antibodies (ACA), three were positive for anti-β2-glycoprotein antibodies IgM, six were positive for antinuclear antibodies(ANAs), four had decreased protein C activity or protein S activity, and three had significantly elevated coagulation factor VIII.

### Radiological examination

**Fig. 1:**
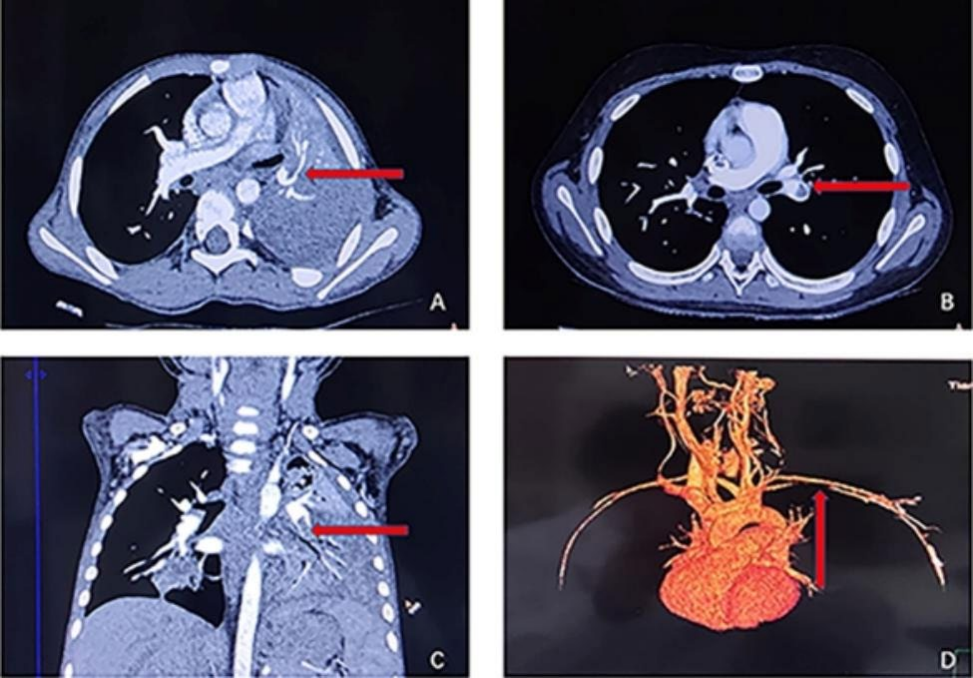
**A**: CTPA revealed consolidation with high density in the left lower lobe and filling defects in the left pulmonary artery. **B**: CTPA showed filling defects in the left pulmonary artery. **C**: Sagittal reconstruction of CTPA showed filling defects in the left pulmonary artery. **D**: CTPA 3D imaging showed a filling defect in the left inferior pulmonary artery

**Fig. 2:**
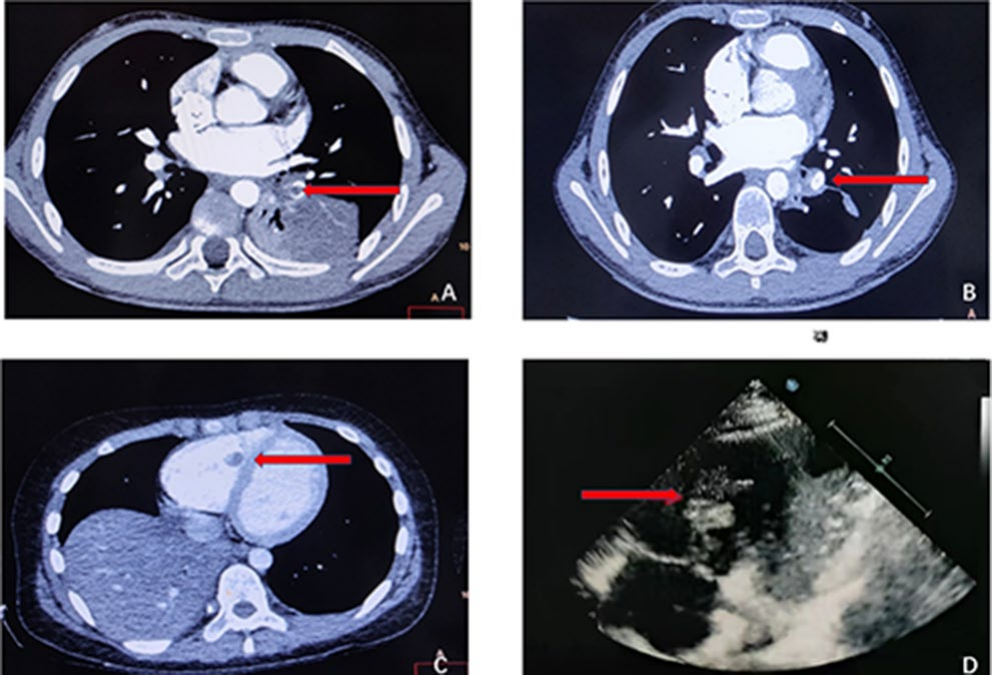
**A**: CTPA showed filling defects in the left pulmonary artery. **B**: Repeat imaging after anticoagulation shows disappearance of filling defect. **C**: CTPA showed filling defects in the right ventricle. **D**: Echocardiography showed mass-like echo in the right ventricle

CT pulmonary angiography (CTPA) was performed on all nine children, revealing filling defects in the corresponding vessels indicative of embolism (as shown in Figs. 1 and 2). Of these cases, three were located in the right lung, three in the left lung, and three in both lungs, with two cases also presenting with cardiac embolism (as depicted in Fig. 2). In addition, all nine children were found to have pleural effusion.

### Treatment

All nine children were treated with antibiotics, with one child receiving doxycycline and eight receiving azithromycin. All children received corticosteroids due to excessive inflammation. Three of the children underwent thoracentesis due to significant pleural effusion. Natriuretic heparin calcium (0.01 ml/Kg) was administered subcutaneously for anticoagulation in nine cases, oral sequential low-molecular heparin anticoagulation with rivaroxaban was given to nine cases, aspirin (5 mg/Kg/d) was used for antiplatelet therapy in six cases, and two cases received combined cardiac and vascular anticoagulation. For thrombolysis in the acute phase, combination urokinase (4400U/Kg/h) was administered to two children with simultaneous cardiac embolisms.

### Outcomes

The outpatient clinic monitored 9 children for a maximum period of 9 months. At the 6-month follow-up, all of the children who had tested positive for aPL showed negative results, and protein S levels returned to normal. After three months, the ANA test results of 6 children who had previously tested positive also became negative. After receiving therapy, all of the children were discharged. Follow-up lung imaging after 1 to 6 months showed a gradual reduction in filling defects, indicating gradual dissipation of the thrombus, and for 2 children who had concomitant heart thrombosis, the thrombus disappeared between 0.5 and 9 months. During the follow-up period, none of the children experienced any recurrence of thrombosis or post-thrombotic pulmonary hypertension.

## Discussion

Accurate epidemiological data on pulmonary embolism (PE) in children is scarce in both the United States and abroad. The early incidence estimates of PE stem from autopsy studies and report an incidence of 0.05–4.2% [8–10]. Studies reported an incidence of 8.6 to 57 per 100,000 in hospitalized children [1, 2], whereas the incidence in all children in the community was estimated to be 0.14 to 0.9 per 100,000 [3, 4]. PE in children has a bimodal age of onset, with two peaks in the neonatal period and adolescence, particularly among those with underlying diseases or risk factors [4]. While rare in children, PE can be life-threatening in severe cases, with a reported mortality rate of 26% [11].

In most cases, Mycoplasma pneumoniae (MP) infections are self-limiting. However, in severe cases, infection can lead to multiple systemic organ complications. Apart from respiratory manifestations, approximately 25% of children with MPP also experience a combination of extrapulmonary symptoms [12]. Narita M has classified extrapulmonary manifestations due to M. pneumoniae infection into three categories [13]: the first is the direct type, in which locally induced cytokines likely play a role; the second is the indirect type, in which immune modulation, such as autoimmunity, is thought to play a role; and the third is the vascular occlusion type, in which vasculitis and/or thrombosis, with or without systemic hypercoagulable states, are believed to play a role.

The specific mechanism of thrombosis caused by MP infection remains unknown, but it may be related to endothelial cell injury, autoimmune response, and an imbalance of coagulation and anticoagulation. The cell membranes of MP contain immune substances such as glycolipids and glycoproteins that share antigens with human tissues and can stimulate the production of autoantibodies. Studies have shown that antiphospholipid antibodies (aPL) and antinuclear antibodies (ANAs) produced by autoimmune reactions caused by MP infection play an important role in thrombosis [14, 15]. Examples of antiphospholipid antibodies include antiphospholipid antibodies (ACA), lupus anticoagulant (LA), and anti-2-glycoprotein antibodies. In Liu’s study of 43 children with MPP and embolism, positive rates for LA were 42.1%, 60% for ACA, 64% for anti-2-glycoprotein antibodies, and 50% for ANAs [16]. In our study, five of nine tested positive for LA, five were positive for ACA, three were positive for anti-β2-glycoprotein antibodies IgM, and six were positive for ANAs. Late follow-up revealed that all antibodies were negative, indicating a potential role for the temporary autoimmune disease brought on by MP infection in the etiology of thrombosis. Moreover, hepatocyte damage caused by MP infection, hypoxia, and immune injury can increase endogenous coagulation factor synthesis [17], while the second synthesis of critical anticoagulation factors such as antithrombin III (AT-III), protein C, and protein S is reduced [18]. Cases of elevated coagulation factors and abnormal anticoagulation proteins have also been reported in MP infection combined with embolism [19–23]. In the current study, three cases had elevated coagulation factor VIII, promoting the body’s hypercoagulable state; three cases had abnormal protein C or protein S activity, preventing the body from performing anticoagulation normally. However, protein C, protein S, and coagulation factor VIII returned to normal during follow-up, indicating a transient imbalance of the coagulation and anticoagulation system caused by MP infection, leading to embolism.

Children who develop pulmonary embolism (PE) may exhibit fewer clinical symptoms than adults and may not display the classic triad of chest pain, hemoptysis, and dyspnea. Unfortunately, diagnosis in children is often delayed, with some studies indicating an average of 7 days from the onset of symptoms to diagnosis [24]. Our study found that PE typically occurred around 2 weeks into the primary illness, and embolic symptoms only emerged in 6 children after their temperature had stabilized and cough had subsided. Furthermore, the time of diagnosis PE did not always coincide with the acute phase of the disease. Early-stage atypical symptoms of PE may be difficult to distinguish from those of primary pulmonary disease, and young children may not be able to accurately report their symptoms. Underlying conditions, such as pneumonia or heart failure exacerbation, can also mask symptoms of PE. As a result, children with abnormal laboratory indicators should be closely monitored for new symptoms, worsening of existing symptoms, or radiating pain at unusual sites, which may indicate the presence of PE.

D-dimer has been proposed as an independent predictor of embolic risk with high sensitivity [25], and a normal level can exclude the possibility of pulmonary embolism [26]. However, D-dimer has low sensitivity and specificity for the diagnosis of PE in children [27]. Although the combination of the Wells score and D-dimer is commonly used to predict PE in adults, and the absence of both factors can rule out PE [28–30], this approach has not been found to be effective in children [28, 29]. Fibrinogen (FIB) can cause increased blood viscosity, which typically occurs 6–15 days after Mycoplasma pneumoniae (MP) infection ^[13]^. This corresponds to the time to embolism in children with MPP in a national study [31]. In our study, all nine children had varying degrees of elevated D-dimer values, and seven had elevated fibrinogen (FIB), with a median time to embolism of approximately 2 weeks from the diagnosis of the MPP. This is consistent with the hypercoagulable state caused by FIB and the time to peak D-dimer levels in the body. Moreover, platelet (PLT) adhesion and aggregation are major mechanisms of PE, and the activation of PLT by the body’s anti-inflammatory mechanism during MP infection, as well as the elevated PLT count in seven cases in our study, is consistent with the results reported by Li et al [32], which suggest that excessive PLT activation may also be a risk factor for PE. Interleukin-6 (IL-6) and C-reactive protein (CRP) are nonspecific markers of inflammation and tissue damage. Studies have shown that high levels of IL-6 and CRP can promote PE [33, 34]. In our study, IL-6 and CRP levels were elevated in all children, respectively. Although elevated levels of IL-6 and CRP can also be caused by various infectious diseases, our study suggests that PE should also be considered in children with these markers.

All nine children in our study were diagnosed with pulmonary embolism (PE) using computed tomography pulmonary angiography (CTPA), a highly sensitive and specific technique that is currently the most common test for confirming the diagnosis of PE [35]. Because of the small size of the emboli and the atypical clinical symptoms in children, embolic signs have sometimes only been detected upon a second review of imaging findings in previous studies [21, 36]. Therefore, particular attention should be paid to the presence of PE during the imaging review of children with MPP who present with symptoms or abnormal laboratory indicators. The presence of thrombus should be given high priority when evaluating imaging results in such children. In an analysis of 18 children with MPP, solid lung changes (> 2/3 of all lobes) and pleural effusion were identified as independent risk factors for embolism [37]. Notably, all of the children in our study had pleural effusion and pulmonary atelectasis.

There are currently no specific management recommendations for pediatric thromboembolism, and treatment protocols are mainly based on clinical experience and evidence-based research in adult patients. Anticoagulation is the primary therapy for children with hemodynamically stable pulmonary embolism (PE), and fast-acting anticoagulants like unfractionated heparin (UFH) and low molecular weight heparin (LMWH) are recommended as initial treatment [38]. After initial parenteral anticoagulation (UFH, LMWH, etc.), oral anticoagulants such as warfarin should be added. During warfarin therapy, the international normalized ratio (INR) should be monitored, and an INR maintained at 2–3 is recommended. It is generally advised that warfarin be used concurrently with UFH or LMWH for more than 5 days and that the INR be maintained at 2–3 for at least 2 days before discontinuing the medication. However, due to their high dietary impact, difficulty in laboratory monitoring, and teratogenicity, vitamin K antagonists have limited use in children [38]. Direct oral anticoagulants (DOACs) such as dabigatran, argatroban, bivalirudin, rivaroxaban, apixaban, and edoxaban, which have stronger anticoagulant activity and lower bleeding risk, have recently emerged as new options for antithrombotic therapy [39]. Studies have shown that rivaroxaban treatment reduces the risk of recurrence and lessens the burden of thrombosis without increasing bleeding when compared to standard anticoagulants [40]. Clinical trials on rivaroxaban in children (EINSTEIN Jr children I through III) have all been completed. When compared to the conventional anticoagulant warfarin, rivaroxaban has a lower economic burden, is easier to administer, has a faster onset of action, and requires no monitoring [41]. In this study, sequential treatment with rivaroxaban and LMWH was used to treat all nine children with thrombosis. During the follow-up period, there were no side effects such as bleeding or liver or kidney damage, and late follow-up revealed gradual thrombus absorption.

Rapid thrombus dissolution and increased survival are two benefits of thrombolytic treatment. For adult patients with hemodynamic instability who do not have any evident contraindications, European guidelines recommend thrombolytic therapy as the first line of treatment [42]. However, there are no established treatment guidelines for pharmacologic thrombolysis in children, hence it is only used in cases of severe thrombosis that causes hemodynamic instability or acute organ insufficiency. Systemic thrombolysis can be used for high-risk PE in children if the healthcare provider is skilled in thrombolysis and there is evidence that it lowers the risk of death and recurring PE in adults with high-risk PE [43]. Systemic thrombolysis is not recommended for adults with intermediate-risk PE due to the increased risk of major bleeding [44]. In contrast, the use of catheter-directed thrombolysis (CDT) in children with intermediate-risk PE has been shown to have a better outcome [45–47], reduce the dose of thrombolytic agents, decrease bleeding-related complications, and may be the first choice [48]. In contrast, Urokinase and recombinant tissue-type fibrinolytic plasminogen activator (rt-PA) are the two most commonly used thrombolytic medications at present. The majority of indications, routes of administration, dosages, and durations of thrombolysis in children are still based on the knowledge gained from adult therapy guidelines. In the present study, urokinase thrombolysis was used for two children with mixed cardiac embolism without any thrombolysis contraindications in order to prevent a large PE caused by multiple thrombi and a large thrombus activity. After thrombolysis, their condition considerably improved.

Children with pulmonary embolism (PE) are at an increased risk of requiring surgical embolectomy. Current European guidelines recommend that surgical embolectomy be reserved for hemodynamically unstable patients who have failed thrombolysis or have contraindications to thrombolysis [42]. However, a growing body of literature supports surgical removal of emboli in submassive pulmonary embolism, which has been shown to reduce the mortality rate to 1.1% in high and intermediate-risk cases [49]. Previous reports have shown that children with moderate pulmonary hypertension who underwent surgical embolectomy did not experience complications during postoperative follow-up [12, 13, 33]. For children with acute PE and contraindications to anticoagulation, the placement of an inferior vena cava filter may be considered, but the available literature on this topic in children is limited, and further research is needed for confirmation.

## Conclusion

The majority of children with RMPP and PE do not have a family history of thrombotic disease or coagulation problems, and the timing of the embolism may not correspond to the disease’s most severe stage. Children with RMPP, elevated D-dimer, positive aPL, and a significant inflammatory response should be monitored for the development of embolism. Anticoagulation with successive low-molecular heparin treatment and rivaroxaban may be considered.

## Data Availability

The datasets used and/or analysed during the current study available from the corresponding author on reasonable request.

## References

[1] Biss TTB, Kahr LR, Chan WH, Williams AK (2008). Clinical features and outcome of pulmonary embolism in children. Br J Haematol.

[2] Andrew MD, Adams M, Ali M, Anderson K, Barnard R, Bernstein D, Brisson M, Cairney L, DeSai B (1994). Venous thromboembolic complications (VTE) in children: first analyses of the Canadian Registry of VTE. Blood.

[3] van Ommen CHH, Büller H, Hirasing HR, Heijmans RA, Peters HS (2001). Venous thromboembolism in childhood: a prospective two-year registry in the Netherlands. J Pediatr.

[4] Stein PDK, Olson F (2004). Incidence of venous thromboembolism in infants and children: data from the National Hospital Discharge Survey. J Pediatr.

[5] Wang ZY, Ye L, Wang J, Liu Y (2019). Monocyte subsets study in children with Mycoplasma pneumoniae pneumonia. Immunol Res.

[6] Rodrigues EM, Silva A, Nunes S (2018). Excess pneumonia and influenza hospitalizations associated with influenza epidemics in Portugal from season 1998/1999 to 2014/2015. Influenza Other Respir Viruses.

[7] Association CM, Subspecialty Group of Respiratory Diseases, The Society of Pediatrics (2013). Editorial Board, Chinese Journal of Pediatrics. Guidelines for management of community acquired pneumonia in children (the revised edition of 2013) (I). Zhonghua Er Ke Za Zhi.

[8] Emery JL (1962). Pulmonary embolism in children. Arch Dis Child.

[9] Buck JR, Connors RH, Coon WW, Weintraub WH, Wesley JR, Coran AG (1981). Pulmonary embolism in children. J Pediatr Surg.

[10] Byard RW, Cutz E (1990). Sudden and unexpected death in infancy and childhood due to pulmonary thromboembolism. An autopsy study. Arch Pathol Lab Med.

[11] Rajpurkar MB, Amankwah T, Martinez EK, Williams D, Van Ommen S, Goldenberg CH (2017). Pulmonary embolism and in situ pulmonary artery thrombosis in paediatrics. A systematic review. Thromb Haemost.

[12] Huang KL, Niu B, Lu SK (2021). Mycoplasma pneumoniae pneumonia complicated by intracardiac thrombosis and pulmonary embolism in a case and review of the literature. J Clin Pediatr.

[13] Narita M (2010). Pathogenesis of extrapulmonary manifestations of Mycoplasma pneumoniae infection with special reference to pneumonia. J Infect Chemother.

[14] Snowden NW, Longson PB, Pumphrey M (1990). Antiphospholipid antibodies and Mycoplasma pneumoniae infection. Postgrad Med J.

[15] Witmer CMS, Shah AP, Raffini SS. LJ: Mycoplasma pneumoniae, splenic infarct, and transient antiphospholipid antibodies: a new association? *Pediatrics* 2007, 119(1):e292-295. 10.1542/peds.2006-1340. 10.1542/peds.2006-1340 17178923

[16] Liu JH, Wu R, Wang R, Xu B, Zhang H, Li Y, Zhao H (2020). Mycoplasma pneumoniae pneumonia associated thrombosis at Beijing Children’s hospital. BMC Infect Dis.

[17] Margetic S (2012). Inflammation and haemostasis. Biochem Med (Zagreb).

[18] Meng T, Wang JC (2006). Observation of changes in plasma endothelin and antithrombin-III in children with Mycoplasma pneumoniae pneumonia. J Practical Med Technol.

[19] Flateau CA, Deman I, Ficko AL, Andriamanantena C, Fontan D, Viant E, Bonnevie E, Rapp L (2013). Aortic thrombus and multiple embolisms during a Mycoplasma pneumoniae infection. Infection.

[20] Pachet AD-P, Moine C, Marlu M, Rubio R, Bost-Bru A (2019). Splenic infarction associated with transient anti-prothrombin antibodies is a rare manifestation of acute Mycoplasma pneumoniae infection. Arch Pediatr.

[21] Brown SMP, Bush S, Cummins A, Davidson D, Buchdahl S (2008). Mycoplasma pneumonia and pulmonary embolism in a child due to acquired prothrombotic factors. Pediatr Pulmonol.

[22] Graw-Panzer KDV, Rao S, Miller S, Lee ST (2009). Venous thrombosis and pulmonary embolism in a child with pneumonia due to Mycoplasma pneumoniae. J Natl Med Assoc.

[23] Su HY, Jin WJ, Zhang HN (2012). Clinical analysis of a case of mycoplasma pneumonia combined with pulmonary embolism. Chin J Pediatr.

[24] Biss TTR, Williams M, van Ommen S, Chan CH, Goldenberg AKC (2018). Recommendations for future research in relation to pediatric pulmonary embolism: communication from the SSC of the ISTH. J Thromb Haemost.

[25] Johnson EDS, Rodgers JC (2019). The D-dimer assay. Am J Hematol.

[26] Kanis JH, Pike CL, Kline J (2018). Diagnostic accuracy of the D-dimer in children. Arch Dis Child.

[27] Hennelly KEB, Monuteuax MN, Hudgins MC, Kua J, Commeree E, Kimia A, Lee R, Kimia EY, Neuman A (2016). Detection of pulmonary embolism in high-risk children. J Pediatr.

[28] Wells PSA, Rodger DR, Ginsberg M, Kearon JS, Gent C, Turpie M, Bormanis AG, Weitz J, Chamberlain J, Bowie M, Barnes D (2000). Derivation of a simple clinical model to categorize patients probability of pulmonary embolism: increasing the models utility with the SimpliRED D-dimer. Thromb Haemost.

[29] Biss TTB, Kahr LR, Chan WH, Williams AK (2009). Clinical probability score and D-dimer estimation lack utility in the diagnosis of childhood pulmonary embolism. J Thromb Haemost.

[30] Ma YW, Liu Y, Ning D, An Z, Wu M, Lin Q (2017). A safe strategy to rule out pulmonary embolism: the combination of the Wells score and D-dimer test: one prospective study. Thromb Res.

[31] Jin WJ, Zhang WX, Zhang HY (2013). Analysis of 23 cases of Mycoplasma pneumonia combined with embolism. China Pediatr Emerg Med.

[32] Li X, Dong LL, Tang Y (2021). Clinical analysis of thrombotic pulmonary embolism complicated by Mycoplasma pneumoniae pneumonia. Chin Clin Med.

[33] Liu N, Ma J, Meng C et al. Clinical analysis of 10 cases of cardiac embolism in children caused by Mycoplasma pneumoniae infection. Chin Clin J practical Pediatr 2021,36(16):1253–6.

[34] Zhang Y, Zhang Z, Wei R, Miao X, Sun S, Liang G, Chu C, Zhao L, Zhu X, Guo Q (2020). IL (Interleukin)-6 contributes to deep vein thrombosis and is negatively regulated by miR-338-5p. Arterioscler Thromb Vasc Biol.

[35] Moore AJE, Wachsmann J, Chamarthy MR, Panjikaran L, Tanabe Y, Rajiah P (2018). Imaging of acute pulmonary embolism: an update. Cardiovasc Diagn Ther.

[36] Saleem S, Berman B (2020). Deep vein thrombosis and pulmonary embolism in the setting of Mycoplasma infection. Case Rep Med.

[37] Han C, Zhang T, Zheng J, Jin P, Zhang Q, Guo W, Xu Y (2022). Analysis of the risk factors and clinical features of Mycoplasma pneumoniae pneumonia with embolism in children: a retrospective study. Ital J Pediatr.

[38] Young G (2017). Anticoagulation Therapies in Children. Pediatr Clin North Am.

[39] Bartholomew JR (2017). Update on the management of venous thromboembolism. Cleve Clin J Med.

[40] Male C, Lensing AWA, Palumbo JS, Kumar R, Nurmeev I, Hege K, Bonnet D, Connor P, Hooimeijer HL, Torres M (2020). Rivaroxaban compared with standard anticoagulants for the treatment of acute venous thromboembolism in children: a randomised, controlled, phase 3 trial. Lancet Haematol.

[41] Sun YZ, Luo P. Research progress on the application of rivaroxaban in thromboembolic diseases in children. Chin Clin J practical Pediatr 2022,37(9):710–3.

[42] Konstantinides SV, Meyer G, Becattini C, Bueno H, Geersing GJ, Harjola VP, Huisman MV, Humbert M, Jennings CS, Jiménez D (2020). 2019 ESC Guidelines for the diagnosis and management of acute pulmonary embolism developed in collaboration with the european respiratory society (ERS). Eur Heart J.

[43] Monagle P, Cuello CA, Augustine C, Bonduel M, Brandão LR, Capman T, Chan AKC, Hanson S, Male C, Meerpohl J (2018). American Society of Hematology 2018 guidelines for management of venous thromboembolism: treatment of pediatric venous thromboembolism. Blood Adv.

[44] Ortel TL, Neumann I, Ageno W, Beyth R, Clark NP, Cuker A, Hutten BA, Jaff MR, Manja V, Schulman S (2020). American Society of Hematology 2020 guidelines for management of venous thromboembolism: treatment of deep vein thrombosis and pulmonary embolism. Blood Adv.

[45] Akam-Venkata J, Forbes TJ, Schreiber T, Kaki A, Elder M, Turner DR, Kobayashi D (2019). Catheter-directed therapy for acute pulmonary embolism in children. Cardiol Young.

[46] Ji D, Gill AE, Durrence WW, Shah JH, Paden ML, Patel KN, Williamson JL, Hawkins CM (2020). Catheter-Directed Pharmacologic Thrombolysis for Acute Submassive and massive pulmonary emboli in children and Adolescents-An exploratory report. Pediatr Crit Care Med.

[47] Belsky J, Warren P, Stanek J, Kumar R (2020). Catheter-directed thrombolysis for submassive pulmonary embolism in children: a case series. Pediatr Blood Cancer.

[48] Ross C, Kumar R, Pelland-Marcotte MC, Mehta S, Kleinman ME, Thiagarajan RR, Ghbeis MB, VanderPluym CJ, Friedman KG, Porras D (2022). Acute Management of High-Risk and Intermediate-Risk Pulmonary Embolism in Children: a review. Chest.

[49] Goldberg JB, Spevack DM, Ahsan S, Rochlani Y, Dutta T, Ohira S, Kai M, Spielvogel D, Lansman S, Malekan R (2020). Survival and right ventricular function after Surgical Management of Acute Pulmonary Embolism. J Am Coll Cardiol.

